# Convolution neural network for the diagnosis of wireless capsule endoscopy: a systematic review and meta-analysis

**DOI:** 10.1007/s00464-021-08689-3

**Published:** 2021-08-23

**Authors:** Kaiwen Qin, Jianmin Li, Yuxin Fang, Yuyuan Xu, Jiahao Wu, Haonan Zhang, Haolin Li, Side Liu, Qingyuan Li

**Affiliations:** 1 Nanfang Hospital (The First School of Clinical Medicine), Southern Medical University, Guangzhou, Guangdong China; 2 Guangzhou SiDe MedTech Co.,Ltd, Guangzhou, Guangdong China; 3 Guangdong Provincial Key Laboratory of Gastroenterology, Department of Gastroenterology, Nanfang Hospital, Southern Medical University, No. 1838, Guangzhou Avenue North, Guangzhou, Guangdong China; 4 State Key Laboratory of Organ Failure Research, Guangdong Provincial Key Laboratory of Viral Hepatitis Research, Department of Hepatology Unit and Infectious Diseases, Nanfang Hospital, Southern Medical University, Guangzhou, Guangdong China

**Keywords:** Deep learning, Convolutional neural network, Capsule endoscopy

## Abstract

**Background:**

Wireless capsule endoscopy (WCE) is considered to be a powerful instrument for the diagnosis of intestine diseases. Convolution neural network (CNN) is a type of artificial intelligence that has the potential to assist the detection of WCE images. We aimed to perform a systematic review of the current research progress to the CNN application in WCE.

**Methods:**

A search in PubMed, SinoMed, and Web of Science was conducted to collect all original publications about CNN implementation in WCE. Assessment of the risk of bias was performed by Quality Assessment of Diagnostic Accuracy Studies-2 risk list. Pooled sensitivity and specificity were calculated by an exact binominal rendition of the bivariate mixed-effects regression model. *I*^2^ was used for the evaluation of heterogeneity.

**Results:**

16 articles with 23 independent studies were included. CNN application to WCE was divided into detection on erosion/ulcer, gastrointestinal bleeding (GI bleeding), and polyps/cancer. The pooled sensitivity of CNN for erosion/ulcer is 0.96 [95% CI 0.91, 0.98], for GI bleeding is 0.97 (95% CI 0.93–0.99), and for polyps/cancer is 0.97 (95% CI 0.82–0.99). The corresponding specificity of CNN for erosion/ulcer is 0.97 (95% CI 0.93–0.99), for GI bleeding is 1.00 (95% CI 0.99–1.00), and for polyps/cancer is 0.98 (95% CI 0.92–0.99).

**Conclusion:**

Based on our meta-analysis, CNN-dependent diagnosis of erosion/ulcer, GI bleeding, and polyps/cancer approached a high-level performance because of its high sensitivity and specificity. Therefore, future perspective, CNN has the potential to become an important assistant for the diagnosis of WCE.

**Supplementary Information:**

The online version contains supplementary material available at 10.1007/s00464-021-08689-3.

Wireless capsule endoscopy (WCE) is a powerful medical instrument for the screening and diagnosis of intestine diseases [1]. According to the clinical practice of ESGE, capsule endoscopy is the first-line investigation in patients with obscure gastrointestinal bleeding [2]. It is also an important tool for the surveillance of Crohn’s disease, polyposis syndromes, and small-bowel cancers [1]. However, the defect of long reading time and the large number of frames restrict the development of WCE [3]. An average of 12,000 images are captured in a single WCE session, and gastroenterologists manually read WCE films with an average reading time of 30–40 min [2, 4].

“Deep learning” is an advanced form of machine learning, which is classified and fed back through multi-layer feature extraction [5]. The most popular learning algorithm for image analysis is the convolutional neural network (CNN) [6]. It can automatically and adaptively learn spatial hierarchies of features through backpropagation by using multiple building blocks [7]. Recently, CNN has attracted great attention and displays great performance in range of image recognition tasks including radiology [8], nephrology [9], and skin lesions [10]. More excitedly, CNN has been successfully used in many clinical applications such as the classification, detection, and segmentation tasks of images in radiology [11]. The diversity of machine learning methods is shown in Fig. 1.

**Fig. 1:**
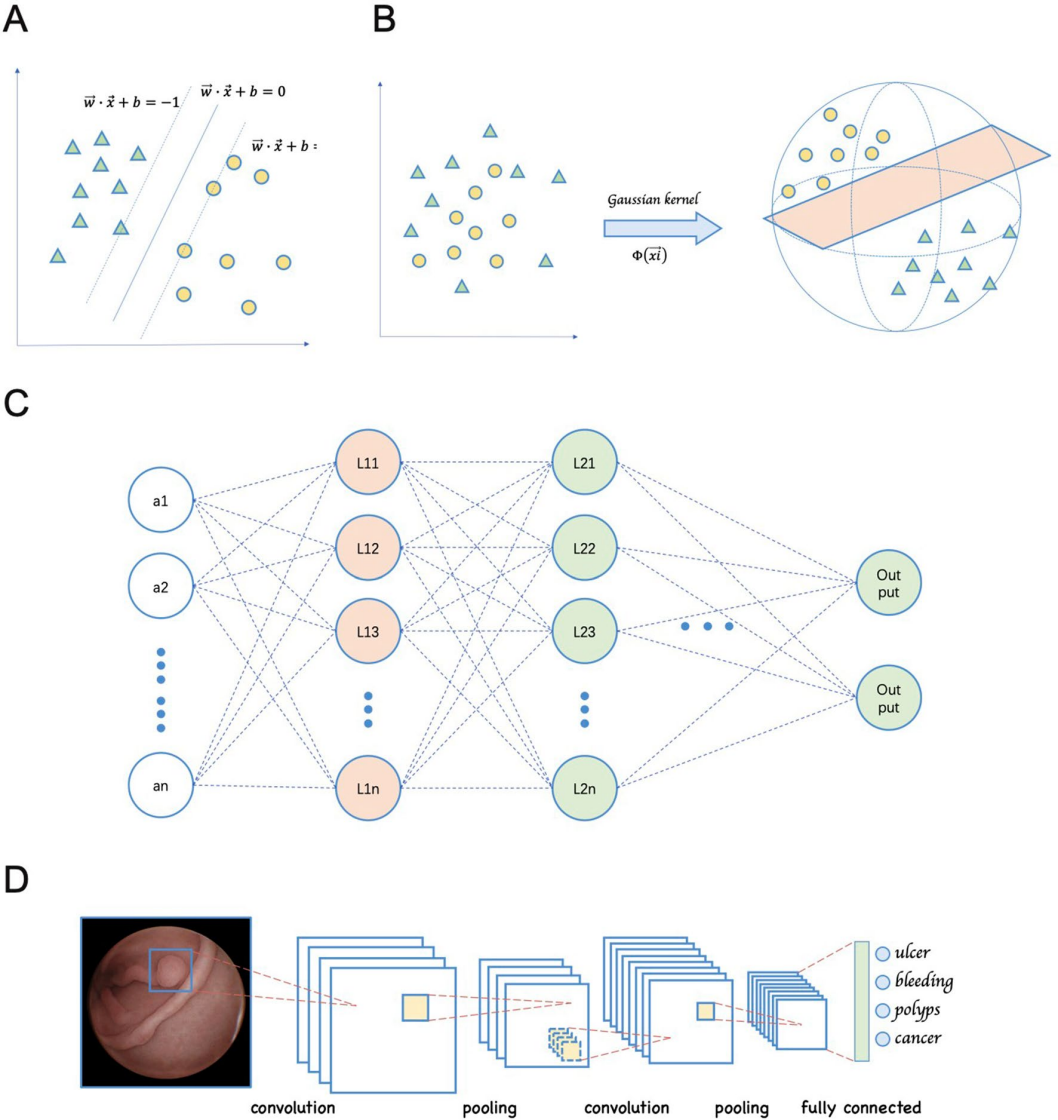
Different kind of machine learning algorithm. **A** Support vector machine (SVM) of linear classification. The data were classified with optimal hyper plane. **B** SVM of non-linear classification. When the data are linearly indivisible, a kernel function is used to map data to high dimensional space. **C** The structure of traditional neural network. An input is passed through the network layers using random weight, by the back propagation, the network fine-tunes the weights based on the error between the calculated output and the actual desired output. However, the large amount of linkage between different nodes of each layers greatly increases the number of parameters and the complexity of the algorithm. **D** Brief schematic diagram of CNN

In the field of endoscopy, the CNN model under supervised learning is the most widely used artificial intelligence (AI) method and is growing mature. The application of it can be divided into two groups: Computer-aided examination and diagnosis [12], which is widely used in the detection of various lesions such as gastric cancer, esophageal cancer, Intestine tumor, and Cokic Polyp, with the sensitivity and specificity which are much higher than experienced physicians [13–17].

Thus, deep learning has the potential to automatically detect diseases and shorten WCE reading time. In this study, we constructed a systematic review and meta-analysis to assess the application and performance of CNN for WCE.

## Materials and methods

We conducted this review in accordance with the Preferred Reporting Items for systematic reviews and meta-analysis (PRISMA2020) [18]. The Grading of Recommendation Assessment, Development, and Evaluation (GRADE) is conducted for assessment of the quality of evidence [19]. The checklist of PRISMA2020 can be approached in Supplementary Table 1.

### Search strategy

We systematically searched studies that assessed the accuracy of CNN for the diagnosis of gastrointestinal diseases by the use of WCE via PubMed, SinoMed, and Web of Science.

The search formula and corresponding search results of each database can be achieved in Supplementary Tables 2 and 3. We searched the databases between Jan 1, 2016, and March 15, 2021. We also searched the reference list of each primary study identified and previous systematic reviews.

### Study selection

Two reviewers (KQ and JL) independently screened the titles and abstracts to determine whether the studies met inclusion criteria. Inclusion was based on titles and abstracts, as well as the full-text article.

Only those studies which are associated with both WCE and CNN can be selected. Primary studies that only use normal endoscopy or did not include the usage of CNN are excluded from our research.

Furthermore, the studies had to provide sufficient information to construct the 2 × 2 contingency table (true and false positives and negatives). Calculation methods of accuracy, specificity, and sensitivity are shown below.

$${\text{Accuracy}} = \frac{{\text{TP + TN}}}{{\text{TP + TN + FP + FN}}},$$

$${\text{Sensitivity}} = \frac{{{\text{TP}}}}{{\text{TP + FN}}},$$

$${\text{Specificity}} = \frac{{{\text{TN}}}}{{\text{TN + FP}}}.$$

We only included publications written in English. Animal experiments, reviews, correspondences, case reports, expert opinions, and editorials were excluded. Disagreements in the inclusion process were resolved by a third reviewer.

### Quality assessment and risk of bias

To evaluate the risk of bias, two reviewers independently applied the Quality Assessment of Diagnostic Accuracy Studies-2 (QUADAS-2) risk checklist [20] for the testing of bias risk of each study. Details of the list can be approached in Supplementary Table 4. Revman 5.4 (Cochrane Collaboration, London, United Kingdom) was used for the assessment of QUADAS-2 and bias risk.

### Data extraction

Data from all included studies were collected into a standardized data extraction sheet, which included the year of publication, study design, application of pathology, type of database, algorithm, capsule brand, training set, validation set, and test set. The investigator also recorded the number of true and false positives and negatives. We contacted the corresponding authors if necessary information was needed.

If a study provides multiple contingency tables related to diagnostic accuracy due to different algorithms, we assume that these contingency tables are independent of each other. We extract the contingency table with the highest overall accuracy into our study for analysis.

### Statistical analysis

A subgroup analysis of the three most frequent types of lesions was performed to evaluate the quality of CNN in WCE image diagnosis, which are respectively erosion/ulcer, GI bleeding, and polyps/cancer. We tabulated true positives, false negatives, false positives, and true negatives of each research, which were used to calculate sensitivity and specificity and a corresponding CI.

To synthesis data, an exact binominal rendition of the bivariate mixed-effects regression model developed by van Houwelingen [21] was used for our analysis. This model does not transform pairs of sensitivity and specificity of individual studies into a single indicator of diagnostic accuracy, but preserves the two-dimensional nature of the data taking into account any correlation between the two [22]. The mean logit sensitivity and specificity with their standard error and 95% CIs, the between-study variability in logit sensitivity and specificity, and covariance between them were estimated based on this model.

The original receiver operating curve scale was used to back-transform these quantities to obtain summary sensitivity, specificity, and diagnostic odds ratios. We then used the derived logit estimates of sensitivity, specificity, and respective variances to construct a hierarchical summary receiver operating curve for CNN with summary operating points for sensitivity and specificity on the curves and a 95% confidence contour ellipsoid. Additionally, the Fagan nomogram was used for the diagnosis of CNN.

*I*^2^ was used to assess heterogeneity, *I*^2^ values > 50% were considered with significant heterogeneity. Study-level covariates can be used in meta-regression to combine results from multiple studies with attention to between-study variation [23]. To investigate publication bias, we constructed Deeks’ funnel plots of asymmetry [24].

MIDAS module for STATA (version 15) was used for the meta-regression and bivariate summary receiver operating curve analysis. Graphs were produced with the MIDAS module and the QUADAS for Revman (version 5.4).

### Certainty assessment

The five GRADE considerations (risk of bias, indirectness, inconsistency, precision, and publication bias) are used to assess the certainty of evidence in our research [19]. We assess our certainty of evidence as high, moderate, low, and very low. GRADE pro GDT software is used for the process of assessment and the preparation of the “Summary of finds” table. We justified all decisions to down- or up-grade the certainty of studies using footnotes.

## Results

**Fig. 2:**
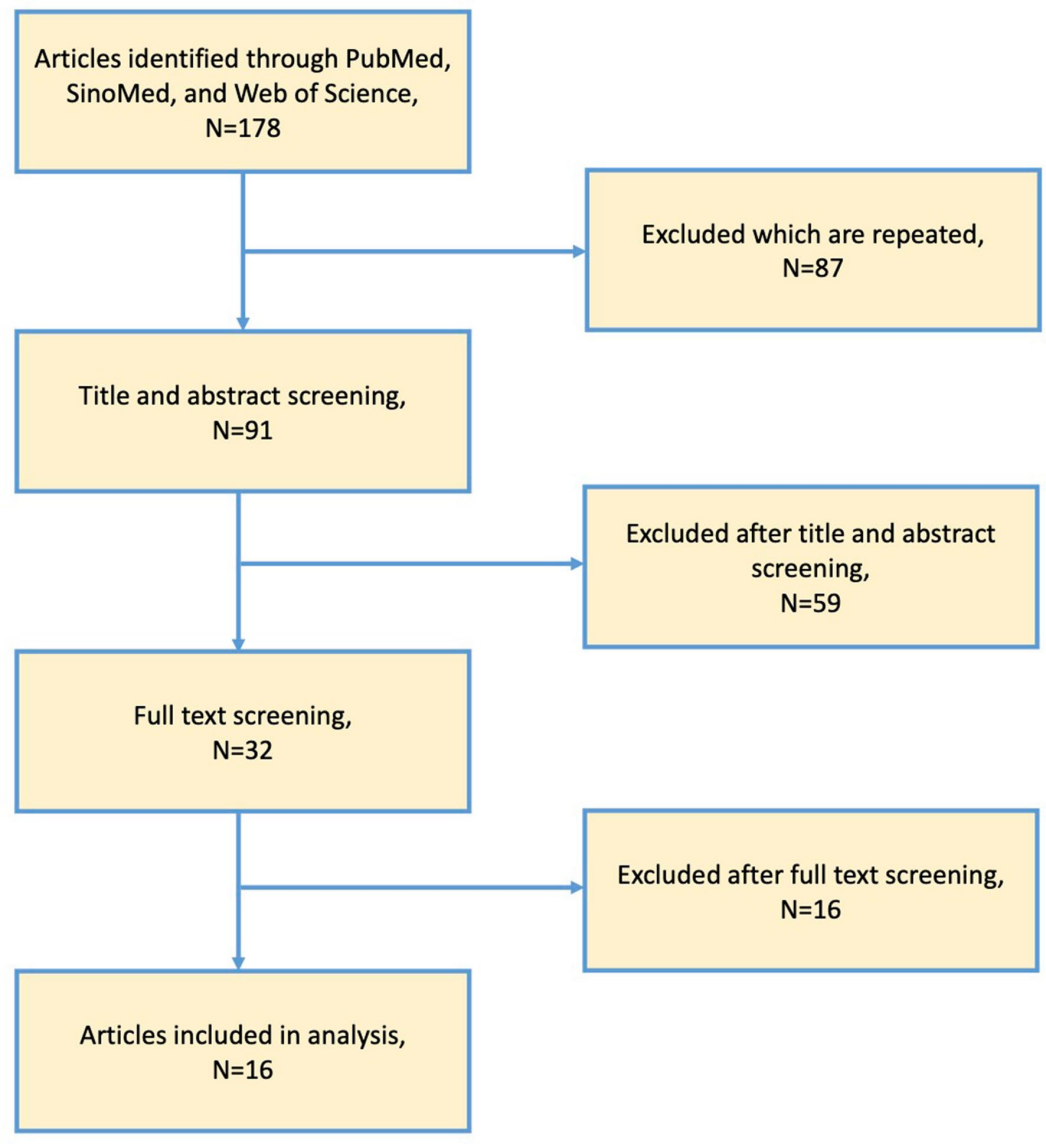
Flow diagram of included studies search of using PubMed, SinoMed, and Web of Science

The database search of us retrieved 178 articles from PubMed, SinoMed, and Web of Science. First of all, 87 repeated articles were excluded. After title and abstract screening, 59 articles were excluded; finally, 16 articles were excluded after the full-text screening, leaving 16 articles with 23 independent studies for inclusion (when two or more lesions appear in one article, we regard them as independent studies.) (Fig. 2).

### Describe summary of the results

In many studies, researchers reported the diagnostic accuracy of different diseases. Thus, we selected the three most representative lesions in the Department of Gastroenterology for subgroup analysis, which are separately ulcer/erosion (nine studies), GI bleeding (seven studies), and polyps/cancer (seven studies). [25–40]

In total, 69,991,447 WCE images in which there are 15,291,964 lesion WCE images were included. All of those studies are retrospective studies. 13 of them use private databases of images while 3 applied online databases.

Most of the studies used CNN for automatic detection, only one of them applied handwork and CNN joint applied. Pillcam and Ankon technology are the two widest used brands of WCE. All the research reported a relatively high accuracy, which is up to 80%, while most of those studies’ sensitivity and specificity are around 90%.

For the detection of ulcers and erosions, Sen Wang et al. [31] used 49,064 images in which there are 24,839 lesion images. An accuracy of 92.1% was achieved. As for the diagnosis of GI bleeding, Aoki et al. [37] reported a 99.9% accuracy with the usage of ResNet-50. In the determination of Polyps, Zhen Ding et al. [33] applied an extensive training set and validation set with 18,068,055 normal images and 5,912,433 polyps’ images and achieve an accuracy of nearly 100%.

The summary of studies that applied CNN techniques for WCE image analysis was listed in Table 1.

**Table 1:** Summary of studies in the literature review that applied CNN techniques for WCE image analysis

Study	Year of publication	Study design	Application	Applied position	Type of database	Algorithm	Capsule brand	Training set	Validation set	Test set	Accuracy (%)	Specificity (%)	Sensitivity (%)	Total dataset size	Images with pathology	TP	FP	FN	TN
Ji Xia [25]	2016	Retrospective	GI bleeding	Small intestine	Proprietary	CNN system based on SVM	–	2050 positive images and 6150 negative images	–	800 positive images and 1000 negative images	99.6	99.2	99.2	1800	800	794	1	6	999
Xiao Jia [26]	2017	Retrospective	GI bleeding	–	Online	CNN and handwork joint classify	–	200 bleeding images and 800 normal images	–	100 bleeding images and 400 normal images	97.2	91	98.8	500	100	91	5	9	395
Yixuan Yuan [27]	2017	Retrospective	Polyps/tumors	Colon and rectum	Proprietary	SSAEIM model	Pillcam SB-WCE	–	–	3000 normal images and 1000 polyps images	98	99	95	4000	1000	950	30	50	2970
Tomonori Aoki [28]	2019	Retrospective	Erosion/ulcer	Small intestine	Proprietary	CNN system based on SSD	Pillcam®SB2/SB3 (Given Imaging)	5360 images of erosion and ulcer from 115 patients between 2009/10 and 2014/12	10,440 independent images from 65 patients between 2015/1 and 2018/1	–	90.8	90.9	84.8	10,440	440	373	913	52	9087
Sen Wang [29]	2019	Retrospective	Ulcer	Gastrointestinal tract	Proprietary	HaNet	Ankon WCE	1416 WCE videos from 30 hospitals and 100 health center collected by Ankon WCE system (70%)	1416 WCE videos from 30 hospitals and 100 health center collected by Ankon WCE system (10%)	1416 WCE videos from 30 hospitals and 100 health center collected by Ankon WCE system (20%)	92.1	90	91.6	49,064	24,839	22,723	1979	2116	22,246
Shanhui Fan [30]	2019	Retrospective	Ulcer	Small intestine	Proprietary	AlexNet	–	2000 ulcer images and 2400 normal images	750 ulcer images and 2000 normal images	500 ulcer images and 600 normal images	95.6	96.8	94.8	21,160	8160	7899	677	261	12,323
Sen Wang [31]	2019	Retrospective	Ulcer	–	Proprietary	RetinaNet	Ankon WCE	15,781 ulcer images and 17,138 normal images	2040 ulcer images and 2319 normal images	4917 ulcer images and 5007 normal images	90.1	90	89.7	9924	4917	4384	477	533	4,530
Victoria Blanes-Vidal [32]	2019	Retrospective	Polyps/tumors	Colon and rectum	Proprietary	AlexNet	PillCam COLON 2	11,300 WCE images which contain 4800 polyps images (70%)	11,300 WCE images which contain 4800 polyps images (15%)	11,300 WCE images which contain 4800 polyps images (15%)	96.4	93.3	97.1	11,300	4,800	4,661	436	139	6,065
Ding [33]	2019	Retrospective	Ulcer	Small intestine	Proprietary	ResNet	Ankon WCE	1970 cases (158,235 images)	5000 cases (113,268,334 images)	–	99.8	99.9	99.7	26,504,010	8,426,916	8,404,163	9039	22,753	18,068,055
			GI bleeding					1970 cases (158,235 images)	5000 cases (113,268,334 images)	–	99.9	99.9	99.5	18,960,561	883,467	879,315	9039	4152	18,068,055
			Polyps/tumors					1970 cases (158,235 images)	5000 cases (113,268,334 images)	–	100	99.9	100	23,989,527	5,912,433	5,911,842	9039	591	18,068,055
Abdul Majid [34]	2020	Retrospective	Ulcer	Stomach	Online	CNN deep feature extraction	–	–	–	2000 ulcer RGB images from Kavsir and 2000 normal RGB images from Private	98.6	99	98	4000	2000	1980	40	20	1960
			GI bleeding					–	–	2000 bleeding RGB images from Private and 2000 normal RGB images from Private	97	98	96	4000	2000	1920	40	80	1960
			Polyps/tumors					–	–	2000 polyps RGB images from Kavsir and 2000 normal RGB images from Private	91.5	98	85	4000	2000	1700	40	300	1960
Eyal Klang [35]	2020	Retrospective	Ulcer	Small intestine	Proprietary	Xception CNN	PillCam SB3	17,640 WCE images from 49 patients (80%)	17,640 WCE images from 49 patients (20%)	-	96.7	96.6	96.8	17,640	7391	17,128	296	512	7095
Ji Xia [36]	2020	Retrospective	Ulcer	Stomach	Proprietary	ResNet-34 CNN	NaviCam MCE(Ankon Technologies, Wuhan, China)	822,590 images from 697 patients in Changhai Hospital between 2014/7 and 2017/12	–	201,365 images from 100 consecutive patients in Changhai Hospital between 2018/1 to 2018/5	93.7	93.7	89.3	136,228	975	121,652	61	14,576	914
			Polyps/tumors					822,590 images from 697 patients in Changhai Hospital between 2014/7 and 2017/12	–	201,365 images from 100 consecutive patients in Changhai Hospital between 2018/1 to 2018/5	94.9	94.8	96.5	137,007	1754	132,212	91	4795	1663
Tomonori Aoki [37]	2020	Retrospective	GI bleeding	Small intestine	Proprietary	ResNet-50 CNN	Pillcam®SB2/SB3 WCE (Given Imaging)	27,847 CE images from Tokyo University Hospital between 2009/11 and 2015/8	–	10,208 CE images from Tokyo University Hospital between 2009/11 to 2015/8	99.9	99.9	96.6	10,208	208	201	4	7	9996
Atsuo Yamada [38]	2020	Retrospective	Polyps/tumors	Colon and rectum	Proprietary	CNN system based on SSD	PillCam COLON 2 WCE (Medtronic, Minneapolis, MN, USA)	156 radom cases (15,933 images) from 178 Patients with colorectal cancer	1850 images from 22 cancer patients and 2934 images from six normal patients	–	83.9	87	79	4784	1850	1462	381	389	2553
Keita Otani [39]	2020	Retrospective	Ulcer	Small intestine	Proprietary	RetinaNet	pillcam SB2/SB3	167 patients’ WCE images from Tokyo Hospital	288 patients’ WCE images from Ishikawa Prefectural Cen-tral Hospital, Fukui Prefectural Hospital, and Tonan Hospital	–	99.4	99.5	92.2	34,835	398	367	172	31	34,265
			GI bleeding								99.3	99.6	78.2	34,975	538	421	138	117	34,299
			Tumors								93.4	94.6	84.7	39,027	4590	3888	1860	702	32,577
Andrea Caroppo [40]	2021	Retrospective	GI bleeding	Small bowel	Online	InceptionV3	MicroCam	–	–	2352 images from KID Dataset2	98.2	97.28	98.7	2352	303	299	56	4	1993

### Quality assessment

**Fig. 3:**
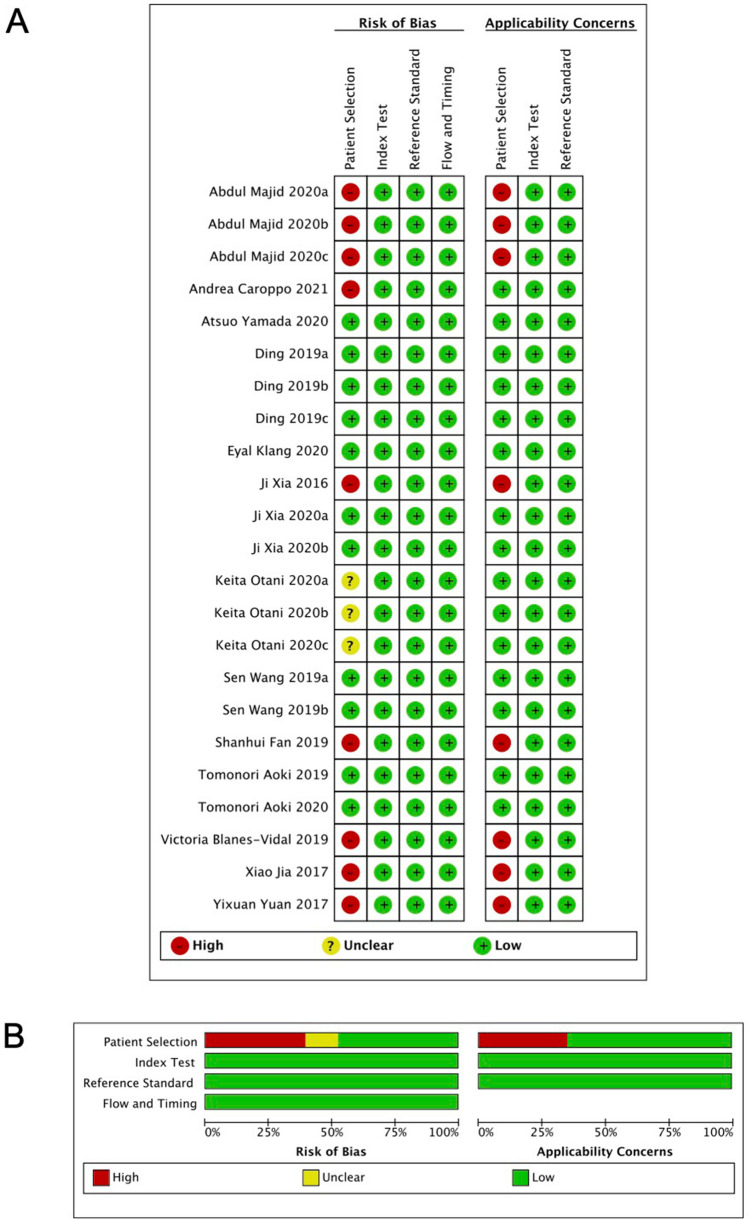
Results of quality assessment. **A** Quality Assessment of Diagnostic Accuracy Studies-2 risk of bias assessment per clinical application. (Abdul Majid 2020a/b/c are respectively ulcer, bleeding and polyps. Ji Xia 2020a/b are respectively ulcer and polyps. Ding 2019a/b/c are respectively ulcer, bleeding, and polyps. Keita Otani 2020a/b/c are respectively ulcer, bleeding, and tumors. Sen Wang 2019a/b are from two different articles, which is cited in Table 1.) **B** Risk of bias and applicability concerns graph: review authors' judgements about each domain presented as percentages across included studies

According to the QUADAS-2 tool, eight of the 23 studies scored a high risk of bias in patient selection, because they did not clearly state the standard of the included images and patients, and we are not sure whether the patients’ sample is a continuous cohort over a period of time. However, all of those studies scored a low risk in index test, reference standard, and flow and timing, which guarantees a low risk of bias. The summary of the quality assessment is presented in Fig. 3.

### Diagnostic accuracy analysis

**Fig. 4:**
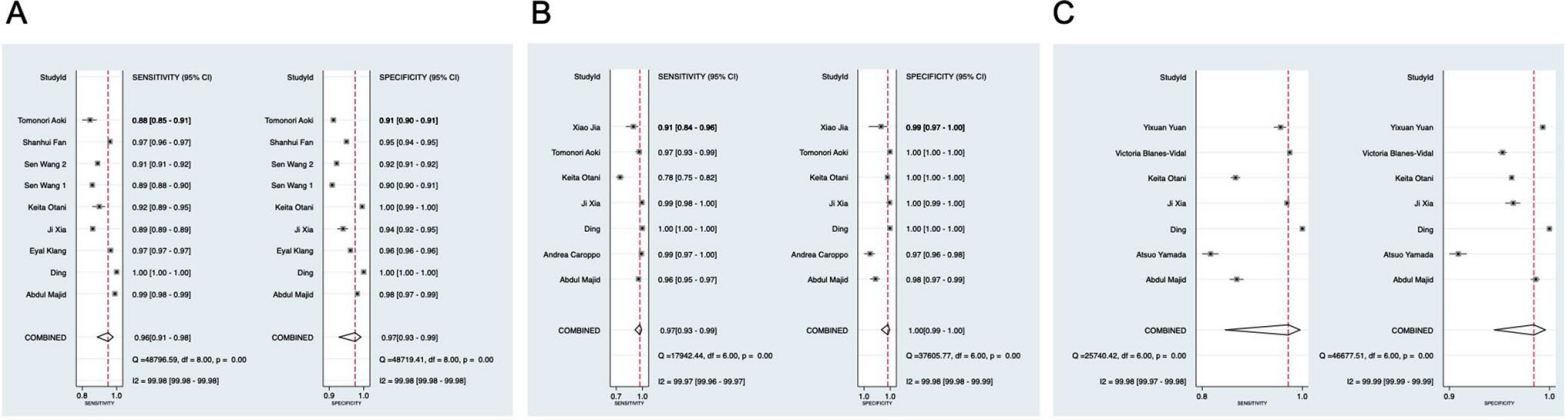
Sensitivity and specificity of included studies as well as their corresponding results. **A** Detection of erosion or ulcer. **B** Detection of GI bleeding. **C** Detection of polyps or cancer

The detection of erosion/ulcer, GI bleeding, and polyps/cancer respectively contains nine, seven, and seven independent studies. Figure 4 shows the sensitivity and specificity of included studies. The pooled sensitivity of CNN for erosion/ulcer is 0.96 (95% CI 0.91, 0.98), for GI bleeding is 0.97 (95% CI 0.93–0.99), and for polyps/cancer is 0.97 (95% CI 0.82–0.99). The corresponding specificity of CNN for erosion/ulcer is 0.97 (95% CI 0.93–0.99), for GI bleeding is 1.00 (95% CI 0.99–1.00), and for polyps/cancer is 0.98 (95% CI 0.92–0.99). (Table 2).

**Table 2:** Results summary of data analysis

	No. of research	Sensitivity	Specificity	PLR	NLR	DOR	ROC area	*I*-square
Erosion/ulcer	9	0.96 [0.91, 0.98]	0.97 [0.93, 0.99]	36.8 [12.3, 110.1]	0.04 [0.02,0.09]	893 [103,5834]	0.99 [0.98–1.00]	100, 95% CI [100–100]
GI bleeding	7	0.97 [0.93, 0.99]	1.00 [0.99, 1.00]	289.4 [80.3, 1043.0]	0.03 [0.01,0.08]	10,291 [1539, 68791]	1.00 [0.99–1.00]	99, 95% CI [99–100]
Polyps/tumors	7	0.97 [0.82, 0.99]	0.98 [0.92, 0.99]	42.7 [11.3, 161.8]	0.03 [0.01,0.21]	1291 [60, 27808]	0.99 [0.98–1.00]	100, 95% CI [100–100]

*PLR* positive likelihood ratio, *NLR* negative likelihood ratio, *DOR* diagnostic odds ratio

**Fig. 5:**
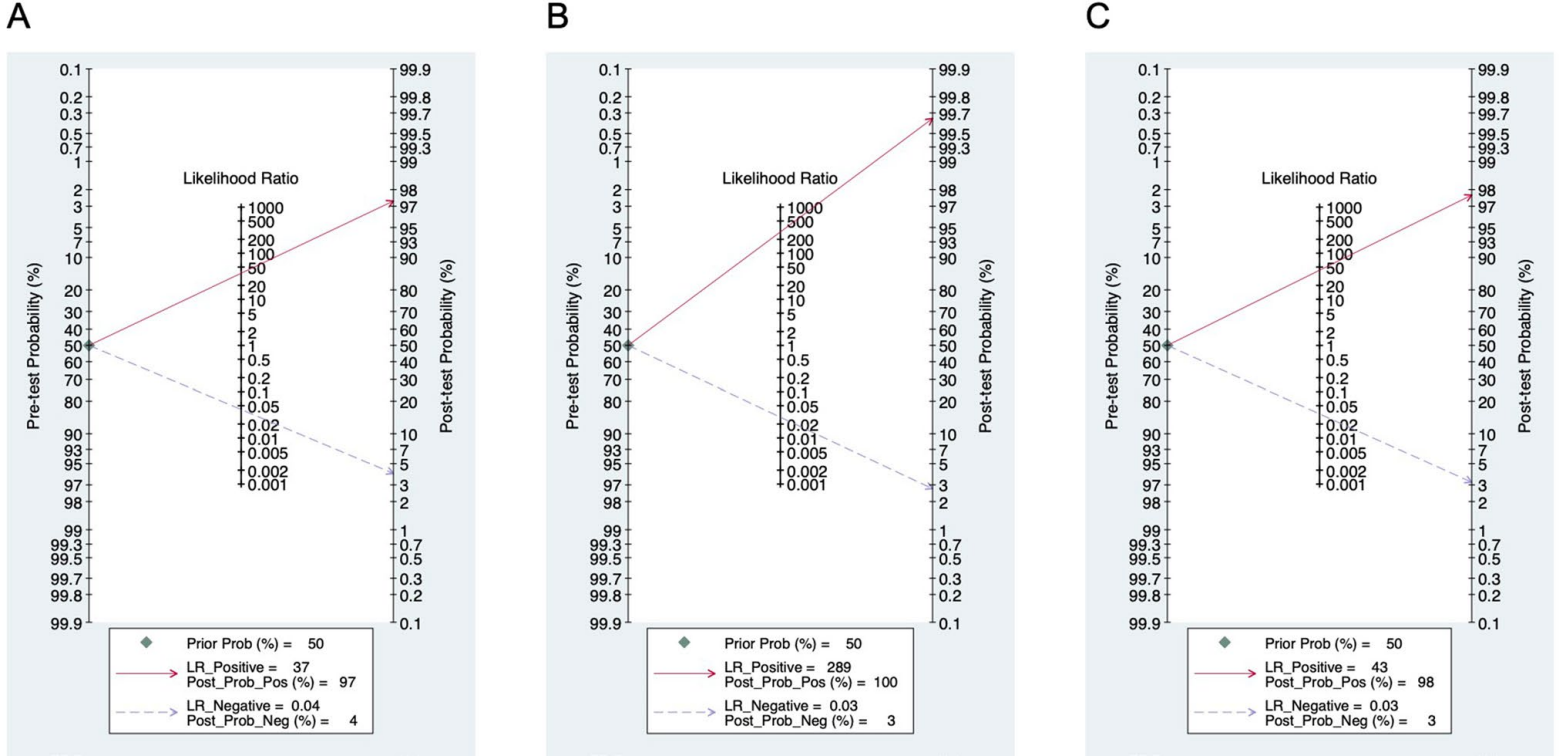
Fagan image of post-test-probability. **A** Erosion or ulcer. **B** GI bleeding. **C** Polyps or cancer

The Fagan images used to describe the post-test probability are shown in Fig. 5. For erosion/ulcer, GI bleeding, and polyps/cancer, the Post-Prob-Pos (posterior probability positive) were separately 0.97, 1.00, and 0.98 while the Post-Prob-Neg (posterior probability negative) were respectively 0.04, 0.03, and 0.03.

Supplementary Fig. 1 presents the bivariate summary receiver operating characteristic (SROC) curves. All subgroups analysis of GI diseases scored a high risk of bias (*p* < 0.1), which were showed in the Deeks’ funnel plot asymmetry test in Supplementary Fig. 2. Substantial heterogeneity exists among the studies (overall *I*^2^ for erosion/ulcer 100%, for GI bleeding 99%, for polyps/cancer 100%). Results summary of data analysis were displayed in Table 2.

“CNN dependent diagnosis of erosion/ulcer, GI bleeding, and polyps/cancer approached a high-level performance.” This evidence downgrades two steps, once for inconsistency and once for publication bias, which results in low test-accuracy in five GRADE considerations. Summary of finds table of each subgroup analysis can be approached separately in Supplementary Tables 5, 6, and 7. The decisions to down- or up-grade the certainty were put in footnotes.

## Discussion

Wireless capsule endoscopy is a technique widely used in the diagnosis of small intestinal diseases [41]. It has been used to detect small intestinal lesions that cannot be reached by traditional endoscopy. At the same time, although it is not the mainstream diagnostic method, capsule endoscopy is also used to explore some esophageal, gastric, and colorectal diseases [42]. Although WCE is convenient and painless, the high rate of miss diagnosis has always been its disadvantage, which is mostly related to people’s limited reading ability and energy [43]. Due to the extension of film reading time, the readers’ attention cannot be focused for a long time, further increasing the misdiagnosis rate [44]. With the development of CNN and the increasing accessibility of public databases, the application of AI in capsule endoscopy has been greatly developed, which reduces the burden of human readers [43].

AI algorithms, especially deep learning algorithms, have made significant progress in image recognition [45]. In the past few years, deep learning has totally changed the field of computer image processing and recognition [11]. Methods from convolutional neural network to variational auto-coding have been widely used in the field of medical image analysis, which promotes the rapid development of medical image analysis [8], and it has been widely accepted that CNN has the power to contribute to the detection of various gastrointestinal diseases including erosion, ulcer, bleeding, polyps, and cancer [46].

So far, the application of CNN in the image diagnosis of WCE has attracted wide attention among the community of endoscopy. An increasing number of CNN research on WCE have been published. Mohan et al. [47] are the first team to conduct a systematic review on the CNN usage in WCE and reported a pooled accuracy of 95.4% on the diagnosis of GI ulcer and hemorrhage. Shelly Soffer et al. [48] also performed a meta-analysis on the CNN detection of ulcer and bleeding. Based on their work, we reviewed the application of CNN in WCE more comprehensively by incorporating more updated studies and included the diagnosis of polyps/cancer lesions.

In our reviews of all studies, we found that CNN acted an excellent ability to the WCE diagnosis of the digestive tract. Almost every research included in our review shows an accuracy of more than 90%, which is comparable with an experienced and senior endoscopist. Besides, high pooled sensitivity and specificity can also be achieved in the diagnosis of ulcer, bleeding, polyps, and cancer. This can indicate the value of clinical practice and reduce the massive and repetitive WCE images needed to be evaluated by human readers [2, 49]. Some research included in our review also compared the performance of CNN with traditional machine learning methods to find the results that CNN was much better than others. For example, Fan’s research indicates that the accuracy of CNN is nearly 25% higher than histogram-SVM for the diagnosis of ulcers [30]. Aoki et al. also report a 20% higher sensitivity on CNN rather than SBI (a conventional tool used to automatically tag images depicting possible bleeding in the reading system) for the detection of bleeding [37]. The gap between CNN and traditional machine learning can be explained by their differences: the diagnosis of machine learning is based on man-selected characteristics such as color, shape, and pattern, which is the simulation of physicians. On the contrary, CNN is a learning system that can automatically extract the features through the training set [50]. However, because of the automatic process of detection, it’s hard for us to find out how the network is constructed. Thus, CNN is often called the “black box” [11].

Besides image detection, CNN also shows great abilities to solve some shortcomings of WCE compared with normal endoscopy. Firstly, before the gastrointestinal examination of capsule endoscopy, diarrhea, and fasting are necessary to clean the gastrointestinal tract. However, it is still difficult to avoid the presence of intestinal contents (such as bile, bubbles, and food residues), which will hinder the observation of mucosa and affect the correct diagnosis and analysis of WCE [51]. Reinier Noorda et al. adopted an automatic evaluation system of capsule endoscopy cleanliness based on a new CNN architecture. By dividing the gastrointestinal tract cleanliness into four grades (poor, general, good, and excellent), the objective and automatic cleanliness evaluation were realized, and a good classification accuracy (95.23%) was achieved [52]. On the field of cleanliness assessment of small-bowel capsule endoscopy, Romain Leenhardt et al. also reported an accuracy of 89.7% to determine whether the bowel preparation is enough or not [53]. Another major problem of capsule endoscopy is the retention at the gastroduodenal junction. Physicians often need to spend several hours observing whether the capsule has entered the duodenum or not [54]. Tao Gan et al. tested a CNN system for automatic detection of capsule endoscopy passing through the gastroduodenal junction, and the probability of judgment time error within 8 min reached 95.7%, which indicate the ability of CNN to help endoscopes automatically determine gastric retention and reduce the time consuming and laborious work [55].

At present, the research of CNN in capsule gastroscopy diagnosis is still limited in the clinical research stage. All the studies are retrospective and most of them only focus on one or two kinds of diseases rather than comprehensive diagnosis. In addition, most of the research data are from single-center, and the clinical research based on multi-center has not been published yet, which will become the direction of our future efforts.

There are some limitations in our study: Firstly, the risk of bias for erosion/ulcers and polyps/cancer is relatively high. It is possibly because the number of cases of lesions like polyps or ulcer is less than that of GI bleeding, while their diagnosis of AI is much more complex, which results in fewer samples of erosion/ulcers, polyps/cancer, and leads to the differences in the data sets used for training and validation in different centers. Secondly, high heterogeneity exists among the studies included in this review, which may be due to the differences of algorithms in some studies, as well as the distinct strictness of experts in different centers for positive judgment of lesions. These limitations result in the low test-accuracy in the certainty assessment of GRADE consideration and may harm the accuracy and applicability of this review.

## Conclusion

In conclusion, our research indicates that CNN has a considerable performance in WCE image diagnosis, and its quality in accuracy, sensitivity, and specificity has reached a relatively high level. With the development of algorithms and computer hardware, the accuracy of CNN will grow higher, and it will become an important tool to help doctors diagnose and play an irreplaceable role in future clinical applications. Besides, research on big data and multi-center will also be the trend of the process of AI application on WCE. Much more data and samples from various patients as training sets are more likely to improve the accuracy and reduce the risk of bias, to achieve the necessary conditions for this technology to be used in routine clinical practice.

## Supplementary Information

Below is the link to the electronic supplementary material.

Supplementary file1 (JPG 127 kb)

Supplementary file2 (JPG 101 kb)

Supplementary file3 (DOCX 47 kb)

## Abbreviations

AI: Intelligence
CNN: Convolution neural network
FN: False negative
FP: False positive
GI: Gastrointestinal
QUADAS-2: Quality assessment of diagnostic accuracy studies-2
SROC: Summary receiver operating characteristic
TN: True negative
TP: True positive
WCE: Wireless capsule endoscopy
