# Supplementary figures and images for: Convolution neural network for the diagnosis of wireless capsule endoscopy: a systematic review and meta-analysis
Source: Surg Endosc. 2021 Aug 23;36(1):16–31. doi: 10.1007/s00464-021-08689-3 (PMC8741689; doi:10.1007/s00464-021-08689-3)

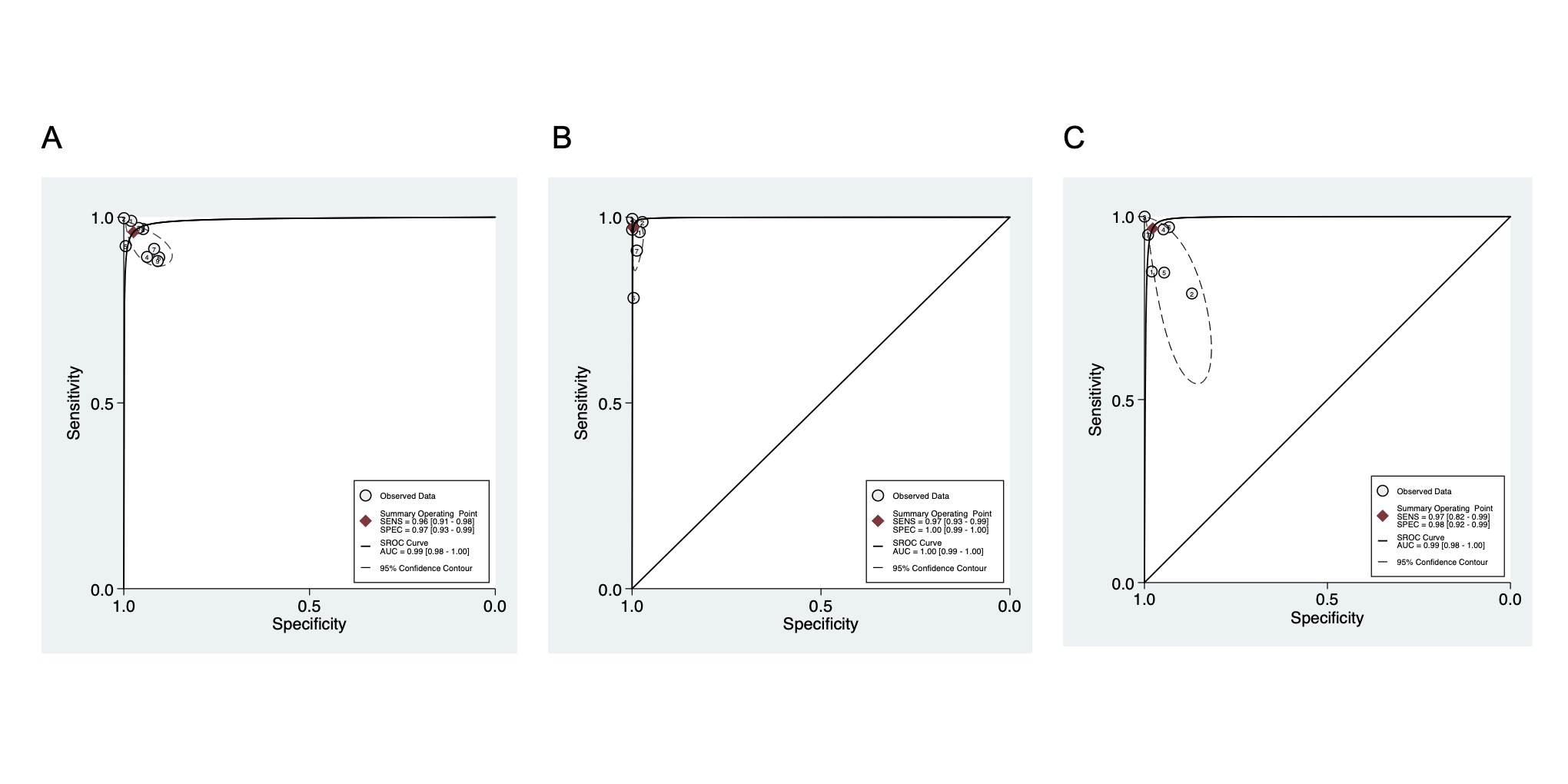

Supplement: Supplementary file 1 — Supplementary file1 (JPG 127 kb) [file 464_2021_8689_MOESM1_ESM.jpg]

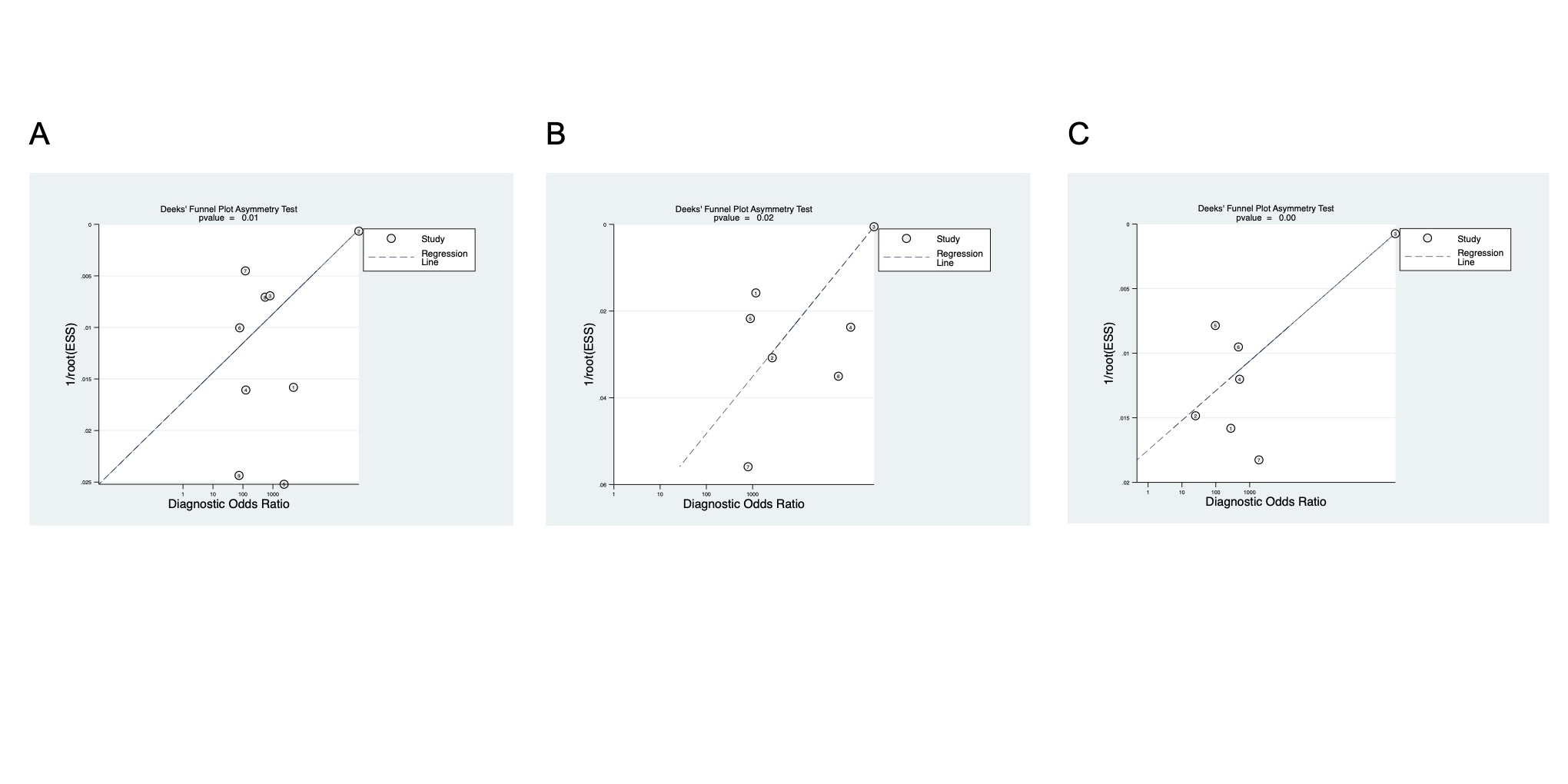

Supplement: Supplementary file 2 — Supplementary file2 (JPG 101 kb) [file 464_2021_8689_MOESM2_ESM.jpg]
