# Supplementary material for: Convolution neural network for the diagnosis of wireless capsule endoscopy: a systematic review and meta-analysis
Source: Surg Endosc. 2021 Aug 23;36(1):16–31. doi: 10.1007/s00464-021-08689-3 (PMC8741689; doi:10.1007/s00464-021-08689-3)
Supplement: Supplementary file 3 — Supplementary file3 (DOCX 47 kb) [file 464_2021_8689_MOESM3_ESM.docx]

Supplementary Table 1. PRISMA 2020 checking list

| No. | Item | Content |
| --- | --- | --- |
| 1 | Title | Convolution neural network for the diagnosis of wireless capsule endoscopy: a systematic review and meta-analysis |
| 2 | abstract | Title: Convolution neural network for the diagnosis of wireless capsule endoscopy: a systematic review and meta-analysis Background: Wireless capsule endoscopy (WCE) is considered to be a powerful instrument for the diagnosis of intestine diseases. Convolution neural network (CNN) is a type of artificial intelligence (AI) that is widely used in the detection of WCE images. We aimed to perform a systematic review of the current research progress to the CNN application in WCE. Methods: A search in PubMed, SinoMed, and Web of Science was conducted to collect all original publications on the subject of CNN implementation in WCE. Assessment of the risk of bias was performed by Quality Assessment of Diagnostic Accuracy Studies-2 (QUADAS-2) risk list. Pooled sensitivity and specificity were calculated with STATA. I2 was used for the evaluation of heterogeneity. Results: 16 articles with 23 independent studies were included. CNN application to WCE was divided into detection on erosion/ulcer, gastrointestinal bleeding (GI bleeding), and polyps/cancer. The pooled sensitivity of CNN for erosion/ulcer is 0.96 [95% CI, 0.91, 0.98], for GI bleeding is 0.97 (95%CI, 0.93-0.99), and for polyps/cancer is 0.97 (95%CI, 0.82-0.99). The corresponding specificity of CNN for erosion/ulcer is 0.97 (95%CI, 0.93-0.99), for GI bleeding is 1.00 (95%CI, 0.99-1.00), and for polyps/cancer is 0.98 (95%CI, 0.92-0.99). Conclusions: Based on our meta-analysis, CNN dependent diagnosis of erosion/ulcer, GI bleeding, and polyps/cancer approached a high-level performance. The quality of evidence is robust because of its high sensitivity and specificity. Therefore, future perspective, CNN has the potential to become an important assistant for the diagnosis of WCE.  Funding: National Natural Science Funds of China (12026605), Guangdong Basic and Applied Basic Research Fund (2020A1515110916), the Guangdong Medical Science and Technology Research Fund Project (A2020143), and the 2020 Southern Medical University Innovation and Entrepreneurship Training Program (202012121035X), the Foundation for the President of Nanfang Hospital of Southern Medical University (2018C027), the Guangdong Science and Technology Plan Project (2017B020209003). |
| 3 | rational | Wireless capsule endoscopy (WCE) is considered to be a powerful instrument for the diagnosis of intestine diseases. Convolution neural network (CNN) is a type of artificial intelligence (AI) that is widely used in the detection of WCE images. |
| 4 | Objectives | We aimed to perform a systematic review of the current research progress to the CNN application in WCE to estimate its sensitivity and specificity |
| 5 | Eligibility criteria | Only those studies which are associated with both WCE and CNN can be selected. Primary studies that only use normal endoscopy or did not include the usage of CNN are excluded from our research.  Furthermore, the studies had to provide sufficient information to construct the 2*2 contingency table (true and false positives and negatives).  We only included publications written in English. Animal experiments, reviews, correspondences, case reports, expert opinions, and editorials were excluded. Disagreements in the inclusion process were resolved by a third reviewer. |
| 6 | Information sources | We systematically searched studies that assessed the accuracy of CNN for the diagnosis of gastrointestinal diseases by the use of WCE via PubMed, SinoMed, and Web of Science. We searched the databases between Jan 1, 2016, and March 15, 2021. |
| 7 | Search strategy | PubMed: ("WCE"[All Fields] OR ("capsule endoscopy"[MeSH Terms] OR ("capsule"[All Fields] AND "endoscopy"[All Fields]) OR "capsule endoscopy"[All Fields] OR ("wireless"[All Fields] AND "capsule"[All Fields] AND "endoscopy"[All Fields]) OR "wireless capsule endoscopy"[All Fields]) OR ("capsule endoscopy"[MeSH Terms] OR ("capsule"[All Fields] AND "endoscopy"[All Fields]) OR "capsule endoscopy"[All Fields]) OR (("wirel commun mob comput"[Journal] OR "wireless"[All Fields]) AND ("endoscopie"[All Fields] OR "endoscopy"[MeSH Terms] OR "endoscopy"[All Fields] OR "endoscopies"[All Fields] OR "endoscopy s"[All Fields]))) AND ("CNN"[All Fields] OR ("deep learning"[MeSH Terms] OR ("deep"[All Fields] AND "learning"[All Fields]) OR "deep learning"[All Fields]) OR (("convolute"[All Fields] OR "convoluted"[All Fields] OR "convolutes"[All Fields] OR "convoluting"[All Fields] OR "convolution"[All Fields] OR "convolutional"[All Fields] OR "convolutions"[All Fields] OR "convolutive"[All Fields]) AND ("neural networks, computer"[MeSH Terms] OR ("neural"[All Fields] AND "networks"[All Fields] AND "computer"[All Fields]) OR "computer neural networks"[All Fields] OR ("neural"[All Fields] AND "network"[All Fields]) OR "neural network"[All Fields]))) AND ("polyp s"[All Fields] OR "polypous"[All Fields] OR "polyps"[MeSH Terms] OR "polyps"[All Fields] OR "polyp"[All Fields] OR ("cancer s"[All Fields] OR "cancerated"[All Fields] OR "canceration"[All Fields] OR "cancerization"[All Fields] OR "cancerized"[All Fields] OR "cancerous"[All Fields] OR "neoplasms"[MeSH Terms] OR "neoplasms"[All Fields] OR "cancer"[All Fields] OR "cancers"[All Fields]) OR ("carcinoma"[MeSH Terms] OR "carcinoma"[All Fields] OR "carcinomas"[All Fields] OR "carcinoma s"[All Fields]) OR ("cysts"[MeSH Terms] OR "cysts"[All Fields] OR "cyst"[All Fields] OR "neurofibroma"[MeSH Terms] OR "neurofibroma"[All Fields] OR "neurofibromas"[All Fields] OR "tumor s"[All Fields] OR "tumoral"[All Fields] OR "tumorous"[All Fields] OR "tumour"[All Fields] OR "neoplasms"[MeSH Terms] OR "neoplasms"[All Fields] OR "tumor"[All Fields] OR "tumour s"[All Fields] OR "tumoural"[All Fields] OR "tumourous"[All Fields] OR "tumours"[All Fields] OR "tumors"[All Fields]) OR ("ulcer"[MeSH Terms] OR "ulcer"[All Fields] OR "ulcerate"[All Fields] OR "ulcerated"[All Fields] OR "ulcerates"[All Fields] OR "ulcerating"[All Fields] OR "ulceration"[All Fields] OR "ulcerations"[All Fields] OR "ulcerative"[All Fields] OR "ulcers"[All Fields] OR "ulcer s"[All Fields] OR "ulcerous"[All Fields]) OR ("erosion"[All Fields] OR "erosions"[All Fields] OR "erosive"[All Fields] OR "erosively"[All Fields] OR "erosivity"[All Fields]) OR ("bleedings"[All Fields] OR "hemorrhage"[MeSH Terms] OR "hemorrhage"[All Fields] OR "bleed"[All Fields] OR "bleeding"[All Fields] OR "bleeds"[All Fields]) OR ("blood vessels"[MeSH Terms] OR ("blood"[All Fields] AND "vessels"[All Fields]) OR "blood vessels"[All Fields] OR "vascular"[All Fields] OR "neovascularization, pathologic"[MeSH Terms] OR ("neovascularization"[All Fields] AND "pathologic"[All Fields]) OR "pathologic neovascularization"[All Fields] OR "vascularisation"[All Fields] OR "vascularization"[All Fields] OR "vascularisations"[All Fields] OR "vascularise"[All Fields] OR "vascularised"[All Fields] OR "vascularities"[All Fields] OR "vascularitis"[All Fields] OR "vascularity"[All Fields] OR "vascularizations"[All Fields] OR "vascularize"[All Fields] OR "vascularized"[All Fields] OR "vascularizes"[All Fields] OR "vascularizing"[All Fields] OR "vasculars"[All Fields]) OR ("lesion"[All Fields] OR "lesion s"[All Fields] OR "lesional"[All Fields] OR "lesions"[All Fields]) OR ("gastrointestinal diseases"[MeSH Terms] OR ("gastrointestinal"[All Fields] AND "diseases"[All Fields]) OR "gastrointestinal diseases"[All Fields]) OR ("GI"[All Fields] AND ("disease"[MeSH Terms] OR "disease"[All Fields] OR "diseases"[All Fields] OR "disease s"[All Fields] OR "diseased"[All Fields])))  SinoMed: (WCE OR wireless endoscopy OR capsule endoscopy) AND (CNN OR Convolutional neural network OR deep learning)  Web of Science: TS = ((WCE OR wireless capsule endoscopy OR capsule endoscopy) AND (CNN OR Convolutional neural network OR deep learning) AND (GI diseases OR Gastrointestinal diseases OR polyps OR ulcer OR cancer OR carcinoma OR bleeding OR GI bleeding OR lesion))  We extracted the search terms from the titles, abstracts and subject headings of two previously published systematic reviews in related fields and developed a search scheme. Search terms were also identified and checked using the PubMed PubReMiner word frequency analysis tool. We also searched the reference list of each primary study identified and previous systematic reviews. |
| 8 | Selection process | Two reviewers independently screened the titles and abstracts to determine whether the studies met inclusion criteria. Inclusion was based on titles and abstract, as well as the full-text article. |
| 9 | Data collection process | Two review authors (KQand JL) extract data from eligible studies. Extracted data were compared, with any discrepancies being resolved through discussion. KQ entered data into Review Manager 5 software (RevMan 5.4), double checking this for accuracy. When information regarding any of the above was unclear, we contacted authors of the reports to provide further details. |
| 10 | Data items | Data from all included studies were collected into a standardized data extraction sheet, which included year of publication, study design, application of pathology, type of database, algorithm, capsule brand, training set, validation set, and test set. The investigator also recorded the number of true and false positives and negatives. We contacted the corresponding authors if necessary information was needed. |
| 11 | Study risk of bias assessment | To evaluate the risk of bias, two reviewers independently applied the Quality Assessment of Diagnostic Accuracy Studies-2 (QUADAS-2) risk check list[1] for the testing of bias risk of each study. Details of the list can be approached in supplementary material 2. Revman 5.4 (Cochrane Collaboration, London, United Kingdom) was used for the assessment of QUADAS-2 and bias risk. Any discrepancies in judgements of risk of bias or justifications for judgements were resolved by discussion to reach consensus between the two review authors, with a third review author acting as an arbiter if necessary. |
| 12 | Effect measures | To estimate the performance of CNN diagnosis for WCE, we tabulated true positives, false negatives, false positives, and true negatives of each research, which were used to calculate sensitivity and specificity and a corresponding 95%CI. |
| 13 | Synthesis methods |  |
| 13a | Describe the processes used to decide which studies were eligible for each synthesis (such as tabulating the study intervention characteristics and comparing against the planned groups for each synthesis | In view of the complexity of the lesions detected by WCE, the two authors independently classify all the lesions mentioned in the studies and found that they are mainly divided into the following categories:  1. erosion 2. ulcer 3. GI bleeding 4. polys 5. cancer Consider the characteristics of the lesions, most studies analyzed ulcers and erosions, tumors and polyps together. Thus, we divide all included studies into 3 subgroups (ulcer/erosion, GI bleeding, polyps/cancer) for synthesis. |
| 13b | Describe any methods required to prepare the data for presentation or synthesis, such as handling of missing summary statistics or data conversions | To synthesis data, an exact binominal rendition of the bivariate mixed-effects regression model developed by van Houwelingen[2] was used for our analysis. The mean logit sensitivity and specificity with their standard error and 95% CIs, the between-study variability in logit sensitivity and specificity, and covariance between them were estimated based on this model. |
| 13c | Describe any methods used to tabulate or visually display results of individual studies and syntheses | The original receiver operating curve scale was used to back-transform these quantities to obtain summary sensitivity, specificity, and diagnostic odds ratios. We then used the derived logit estimates of sensitivity, specificity, and respective variances to construct a hierarchical summary receiver operating curve for CNN with summary operating points for sensitivity and specificity on the curves and a 95% confidence contour ellipsoid. Additionally, the Fagan nomogram was used for the diagnosis of CNN. MIDAS module for STATA (version 15) was used for the meta-regression and bivariate summary receiver operating curve analysis. Graphs were produced with the MIDAS module and the QUADAS for Revman (version 5.4). |
| 13d | Describe any methods used to synthesise results and provide a rationale for the choice(s). If meta-analysis was performed, describe the model(s), method(s) to identify the presence and extent of statistical heterogeneity, and software package(s) used | To synthesis data, an exact binominal rendition of the bivariate mixed-effects regression model developed by van Houwelingen[2] was used for our analysis. This model does not transform pairs of sensitivity and specificity of individual studies into a single indicator of diagnostic accuracy but preserves the two-dimensional nature of the data taking into account any correlation between the two. I2 was used to assess heterogeneity, I2 values >50% were considered with significant heterogeneity. |
| 13e | Describe any methods used to explore possible causes of heterogeneity among study results (such as subgroup analysis, meta-regression) | A subgroup analysis of the three most frequent types of lesions was performed to evaluate the quality of CNN in WCE image diagnosis, which are respectively erosion/ulcer, GI bleeding, and polyps/cancer. |
| 13f | Describe any sensitivity analyses conducted to assess robustness of the synthesised results | Sensitivity analyses were not pre-specified |
| 14 | Reporting bias assessment | To investigate publication bias, we constructed Deeks’ funnel plots of asymmetry，p<0.1 is viewed as high risk of bias.[3] |
| 15 | Certainty assessment | The 5 GRADE considerations (risk of bias, indirectness, inconsistency, precision, and publication bias)[4] are used to assess the certainty of evidence in our research. We assess our certainty of evidence as high, moderate, low, and very low. GRADE pro GDT software is used for the process of assessment and the preparation of "Summary of finds" table. We justified all decisions to down- or up-grade the certainty of studies using footnotes. |
| 16 | Study selection |  |
| 16a | Describe the results of the search and selection process, from the number of records identified in the search to the number of studies included in the review, ideally using a flow diagram | The database search of us retrieved 178 articles from PubMed, SinoMed, and Web of Science. First of all. 87 repeated articles were excluded. After title and abstract screening, 59 articles were excluded; finally, 16 articles were excluded after full-text screening, leaving 16 articles with 23 independent studies for inclusion (when two or more lesions appears in one article, we regard them as independent studies.) |
| 16b | Cite studies that might appear to meet the inclusion criteria, but which were excluded, and explain why they were excluded | Four studies might have been included in our research but finally were excluded. Jun-Yan He et al[5] and Muhammad Attique Khan et al[6] separately study the performance in hookworm detection and stomach deformities recognition, but we can hardly find any similar study which has the same lesion to perform the synthesis. The work of Toshiaki Hirasawa et al[7] and Gregor Urban et al[8] is also eliminated because they don't use endoscopic images instead of capsule endoscopy. |
| 17 | Study characteristics | Data from all included studies were collected into a standardized data extraction sheet, which included year of publication, study design, application of pathology, type of database, algorithm, capsule brand, training set, validation set, and test set. The investigator also recorded the number of true and false positives and negatives. All these characteristics were listed in table 1 |
| 18 | Risk of bias in studies | According to QUADAS-2 tool, 8 of the 23 studies scored a high risk of bias in patient selection, because they didn’t clearly state the standard of the included images and patients, and we are not sure whether the patients’ sample is a continuous cohort over a period of time. However, all of those studies scored a low risk in index test, reference standard, and flow and timing, which guarantees a low risk of bias. The summary of the quality assessment is presented in Figure 3. |
| 19 | Results of individual studies | The detection of erosion/ulcer, GI bleeding, and polyps/cancer respectively contains 9, 7, and 7 independent studies. Figure 4 shows the sensitivity and specificity of each included studies, together with their 95% confidence interval. |
| 20 | Results of syntheses |  |
| 20a | For each synthesis, briefly summaries the characteristics and risk of bias among contributing studies | According to QUADAS-2 tool, 8 of the 23 studies scored a high risk of bias in patient selection, because all of them didn’t clearly state the standard of the included images and patients, and we are not sure whether the patients’ sample is a continuous cohort over a period of time. |
| 20b | Present results of all statistical syntheses conducted. If meta-analysis was done, present for each the summary estimate and its precision (such as confidence/credible interval) and measures of statistical heterogeneity. If comparing groups, describe the direction of the effect | The pooled sensitivity of CNN for erosion/ulcer is 0.96 [95% CI, 0.91, 0.98], for GI bleeding is 0.97 (95%CI, 0.93-0.99), and for polyps/cancer is 0.97 (95%CI, 0.82-0.99). The corresponding specificity of CNN for erosion/ulcer is 0.97 (95%CI, 0.93-0.99), for GI bleeding is 1.00 (95%CI, 0.99-1.00), and for polyps/cancer is 0.98 (95%CI, 0.92-0.99). (Overall I2 for erosion/ulcer 100%, for GI bleeding 99%, for polyps/cancer 100%). |
| 20c | Present results of all investigations of possible causes of heterogeneity among study results | High heterogeneity exists among the studies included in this review, which may be due to the differences of algorithms in some studies, as well as the distinct strictness of experts in different centers for positive judgment of lesions. |
| 20d | Present results of all sensitivity analyses conducted to assess the robustness of the synthesized results | Sensitivity analyses were not pre-specified |
| 21 | Risk of reporting biases in syntheses | According to Deeks' Funnel Plot Asymmetry Test, all subgroup analysis of GI diseases scores a high risk of bias(p<0.1). |
| 22 | Certainty of evidence | CNN dependent diagnosis of erosion/ulcer, GI bleeding, and polyps/cancer approached a high-level performance. This evidence downgrades 2 steps, once for inconsistency and once for publication bias, which results in low test accuracy in 5 GRADE consideration. Suammary of finds table of each subgroup analysis can be apporached in supplementary analysis. the decisions to down- or up- grade the certainty were put in footnotes. |
| 23 | Discussion |  |
| 23a | Provide a general interpretation of the results in the context of other evidence | CNN acted an excellent ability to the WCE diagnosis of digestive tract. Almost every research included in our review shows an accuracy of more than 90%, which is comparable with an experienced and senior endoscopist. Besides, high pooled sensitivity and specificity can also be achieved in the diagnosis of ulcer, bleeding, polyps, and cancer. |
| 23b | Discuss any limitations of the evidence included in the review | At present, the research of CNN in capsule gastroscopy diagnosis is still limited in the clinical research stage. All the studies are retrospective and most of them only focus on one or two kinds of diseases rather than comprehensive diagnosis. In addition, most of the research data are from single center, which will probably affect the accuracy of the results. |
| 23c | Discuss any limitations of the review processes used | Because of time constraints, only 32 articles are conducted full-text screening, for the rest, we review their title and abstract, only one author (KQ) double checks the accuracy of the data, which will induce some risk of error. Finally, only articles written in English are included in our review. |
| 23d | Discuss implications of the results for practice, policy, and future research | With the development of algorithm and computer hardware, the accuracy of CNN will grow higher, and it will become an important tool to help doctors diagnose and play an Irreplaceable role in future clinical application. Besides, research on big data and multi-center will also be the trend of the process of AI application on WCE. Much more data and samples from various patients as training sets are more likely to improve the accuracy and reduce the risk of bias, to achieve the necessary conditions for this technology to be used in routine clinical practice. |
| 24 | Registration and protocol |  |
| 24a | Provide registration information for the review, including register name and registration number, or state that the review was not registered | This systematic review has not registered yet. |
| 24b | Indicate where the review protocol can be accessed, or state that a protocol was not prepared | A protocol was not prepared. |
| 24c | Describe and explain any amendments to information provided at registration or in the protocol | There are no amendments to information provided at registration or in the protocol. |
| 25 | Support | This work was supported by the National Natural Science Funds of China (12026605), Guangdong Basic and Applied Basic Research Fund (2020A1515110916), the Guangdong Medical Science and Technology Research Fund Project (A2020143), and the 2020 Southern Medical University Innovation and Entrepreneurship Training Program (202012121035X), the Foundation for the President of Nanfang Hospital of Southern Medical University (2018C027), the Guangdong Science and Technology Plan Project (2017B020209003). |
| 26 | Competing interests | Drs. Kaiwen Qin, Jianmin Li, Yuxin Fang, Yuyuan Xu, Jiahao Wu, Haonan Zhang, Haolin Li, Side Liu, and Qingyuan Li have no conflicts of interest or financial ties to disclose. |
| 27 | Availability of data, code, and other materials | All meta-analytic data are publicly available at studies on PubMed (https://pubmed.ncbi.nlm.nih.gov/), Web of Science (http://apps.webofknowledge.com/), and SinoMed (http://www.sinomed.ac.cn/). All the code and script can be obtained by contacting the author's (KQ) email (qinkaiwen17@163.com) |

Supplementary Table 2. Corresponding search results of PubMed, SinoMed, and Web of Science.

| **Database** | **Amount of searching results** |
| --- | --- |
| PubMed | 57 |
| SinoMed | 8 |
| Web of Science | 113 |

Supplementary Table 3. Search formula of 3 databases.

| **Database** | **Search strategy** |
| --- | --- |
| PubMed | ("WCE"[All Fields] OR ("capsule endoscopy"[MeSH Terms] OR ("capsule"[All Fields] AND "endoscopy"[All Fields]) OR "capsule endoscopy"[All Fields] OR ("wireless"[All Fields] AND "capsule"[All Fields] AND "endoscopy"[All Fields]) OR "wireless capsule endoscopy"[All Fields]) OR ("capsule endoscopy"[MeSH Terms] OR ("capsule"[All Fields] AND "endoscopy"[All Fields]) OR "capsule endoscopy"[All Fields]) OR (("wirel commun mob comput"[Journal] OR "wireless"[All Fields]) AND ("endoscopie"[All Fields] OR "endoscopy"[MeSH Terms] OR "endoscopy"[All Fields] OR "endoscopies"[All Fields] OR "endoscopy s"[All Fields]))) AND ("CNN"[All Fields] OR ("deep learning"[MeSH Terms] OR ("deep"[All Fields] AND "learning"[All Fields]) OR "deep learning"[All Fields]) OR (("convolute"[All Fields] OR "convoluted"[All Fields] OR "convolutes"[All Fields] OR "convoluting"[All Fields] OR "convolution"[All Fields] OR "convolutional"[All Fields] OR "convolutions"[All Fields] OR "convolutive"[All Fields]) AND ("neural networks, computer"[MeSH Terms] OR ("neural"[All Fields] AND "networks"[All Fields] AND "computer"[All Fields]) OR "computer neural networks"[All Fields] OR ("neural"[All Fields] AND "network"[All Fields]) OR "neural network"[All Fields]))) AND ("polyp s"[All Fields] OR "polypous"[All Fields] OR "polyps"[MeSH Terms] OR "polyps"[All Fields] OR "polyp"[All Fields] OR ("cancer s"[All Fields] OR "cancerated"[All Fields] OR "canceration"[All Fields] OR "cancerization"[All Fields] OR "cancerized"[All Fields] OR "cancerous"[All Fields] OR "neoplasms"[MeSH Terms] OR "neoplasms"[All Fields] OR "cancer"[All Fields] OR "cancers"[All Fields]) OR ("carcinoma"[MeSH Terms] OR "carcinoma"[All Fields] OR "carcinomas"[All Fields] OR "carcinoma s"[All Fields]) OR ("cysts"[MeSH Terms] OR "cysts"[All Fields] OR "cyst"[All Fields] OR "neurofibroma"[MeSH Terms] OR "neurofibroma"[All Fields] OR "neurofibromas"[All Fields] OR "tumor s"[All Fields] OR "tumoral"[All Fields] OR "tumorous"[All Fields] OR "tumour"[All Fields] OR "neoplasms"[MeSH Terms] OR "neoplasms"[All Fields] OR "tumor"[All Fields] OR "tumour s"[All Fields] OR "tumoural"[All Fields] OR "tumourous"[All Fields] OR "tumours"[All Fields] OR "tumors"[All Fields]) OR ("ulcer"[MeSH Terms] OR "ulcer"[All Fields] OR "ulcerate"[All Fields] OR "ulcerated"[All Fields] OR "ulcerates"[All Fields] OR "ulcerating"[All Fields] OR "ulceration"[All Fields] OR "ulcerations"[All Fields] OR "ulcerative"[All Fields] OR "ulcers"[All Fields] OR "ulcer s"[All Fields] OR "ulcerous"[All Fields]) OR ("erosion"[All Fields] OR "erosions"[All Fields] OR "erosive"[All Fields] OR "erosively"[All Fields] OR "erosivity"[All Fields]) OR ("bleedings"[All Fields] OR "hemorrhage"[MeSH Terms] OR "hemorrhage"[All Fields] OR "bleed"[All Fields] OR "bleeding"[All Fields] OR "bleeds"[All Fields]) OR ("blood vessels"[MeSH Terms] OR ("blood"[All Fields] AND "vessels"[All Fields]) OR "blood vessels"[All Fields] OR "vascular"[All Fields] OR "neovascularization, pathologic"[MeSH Terms] OR ("neovascularization"[All Fields] AND "pathologic"[All Fields]) OR "pathologic neovascularization"[All Fields] OR "vascularisation"[All Fields] OR "vascularization"[All Fields] OR "vascularisations"[All Fields] OR "vascularise"[All Fields] OR "vascularised"[All Fields] OR "vascularities"[All Fields] OR "vascularitis"[All Fields] OR "vascularity"[All Fields] OR "vascularizations"[All Fields] OR "vascularize"[All Fields] OR "vascularized"[All Fields] OR "vascularizes"[All Fields] OR "vascularizing"[All Fields] OR "vasculars"[All Fields]) OR ("lesion"[All Fields] OR "lesion s"[All Fields] OR "lesional"[All Fields] OR "lesions"[All Fields]) OR ("gastrointestinal diseases"[MeSH Terms] OR ("gastrointestinal"[All Fields] AND "diseases"[All Fields]) OR "gastrointestinal diseases"[All Fields]) OR ("GI"[All Fields] AND ("disease"[MeSH Terms] OR "disease"[All Fields] OR "diseases"[All Fields] OR "disease s"[All Fields] OR "diseased"[All Fields]))) |
| SinoMed | (WCE OR wireless endoscopy OR capsule endoscopy) AND (CNN OR Convolutional neural network OR deep learning) |
| Web of Science | TS = ((WCE OR wireless capsule endoscopy OR capsule endoscopy) AND (CNN OR Convolutional neural network OR deep learning) AND (GI diseases OR Gastrointestinal diseases OR polyps OR ulcer OR cancer OR carcinoma OR bleeding OR GI bleeding OR lesion)) |

Supplementary Table 4. Quality Assessment of Diagnostic Accuracy Studies-2 checking list.

| **Question** | **Answer** |
| --- | --- |
| **Patient election** | |
| Was a consecutive or random sample of patients enrolled? | Yes/No/Unclear |
| Was a case-control design avoided? | Yes/No/Unclear |
| Did the study avoid inappropriate exclusions? | Yes/No/Unclear |
| **Could the selection of patients have introduced bias?** | **High/Low/Unclear risk** |
| Are there concerns that the included patients and setting do not match the review question? | High/Low/Unclear concern |
| **Index Test** | |
| Were the index test results interpreted without knowledge of the results of the reference standard? | Yes/No/Unclear |
| If a threshold was used, was it pre-specified? | Yes/No/Unclear |
| **Could the conduct or interpretation of the index test have introduced bias?** | **High/Low/Unclear risk** |
| Are there concerns that the index test, its conduct, or interpretation differ from the review question? | High/Low/Unclear concern |
| **Reference Standard** | |
| Are the reference standards likely to correctly classify the target condition? | Yes/No/Unclear |
| Were the reference standard results interpreted without knowledge of the results of the index tests? | Yes/No/Unclear |
| **Could the reference standard, its conduct, or its interpretation have introduced bias?** | **High/Low/Unclear risk** |
| Are there concerns that the target condition as defined by the reference standard does not match the question? | High/Low/Unclear concern |
| **Flow and Timing** | |
| Was there an appropriate interval between index test and reference standard? | Yes/No/Unclear |
| Did all patients receive the same reference standard? | Yes/No/Unclear |
| Were all patients included in the analysis? | Yes/No/Unclear |
| **Could the patient flow have introduced bias?** | **High/Low/Unclear risk** |

Supplementary Table 5. Certainty assessment of GRADE consideration in ulcer/erosion.

| Outcomes | №; studies (№; patients) | Research design | Factors that may decrease certainty of evidence | | | | | Effect per 100 patients tested | Test accuracy CoE |
| --- | --- | --- | --- | --- | --- | --- | --- | --- | --- |
|  |  |  | Risk of bias | Indirectness | Inconsistency | precision | Publication bias | Pre-test probability of ulcer/erosion % |  |
| **True positive** (Patients diagnosed with ulcer/erosion) | 9 studies 8621523 patients | Case-control Study | Not serious | Not serious | Serious^[[1]](#footnote-1)^ | Not serious | Publication bias is strongly suspected^[[2]](#footnote-2)^ | 96 (91 to 98) | ⨁⨁◯◯ LOW |
| **False negative** (Patients misdiagnosed as having no ulcer/erosion) |  |  |  |  |  |  |  | 4 (2 to 9) |  |
| **True negative** (People without ulcers/erosion) | 9 studies 18174129 patients | Case-control Study | Not serious | Not serious | Serious^[[3]](#footnote-3)^ | Not serious | Publication bias is strongly suspected^[[4]](#footnote-4)^ | 97 (93 to 99) | ⨁⨁◯◯ LOW |
| **False positive** (Patients misdiagnosed as ulcer/erosion) |  |  |  |  |  |  |  | 3 (1 to 7) |  |

Supplementary Table 6. Certainty assessment of GRADE consideration in GI bleeding.

| Outcomes | №; studies (№; patients) | Research design | Factors that may decrease certainty of evidence | | | | | Effect per 100 patients tested | Test accuracy CoE |
| --- | --- | --- | --- | --- | --- | --- | --- | --- | --- |
|  |  |  | Risk of bias | Indirectness | Inconsistency | precision | Publication bias | Pre-test probability of ulcer/erosion % |  |
| **True positive** (Patients diagnosed with GI bleeding) | 7 studies 887416 patients | Case-control Study | Not serious | Not serious | Serious^[[5]](#footnote-5)^ | Not serious | Publication bias is strongly suspected^[[6]](#footnote-6)^ | 97 (93 to 99) | ⨁⨁◯◯ LOW |
| **False negative** (Patients misdiagnosed as having no GI bleeding) |  |  |  |  |  |  |  | 3 (1 to 7) |  |
| **True negative** (People without GI bleeding) | 7 studies 18126980 patients | Case-control Study | Not serious | Not serious | Serious^[[7]](#footnote-7)^ | Not serious | Publication bias is strongly suspected^[[8]](#footnote-8)^ | 100 (99 to 100) | ⨁⨁◯◯ LOW |
| **False positive** (Patients misdiagnosed as GI bleeding) |  |  |  |  |  |  |  | 0 (0 to 1) |  |

Supplementary Table 7. Certainty assessment of GRADE consideration in polyps/cancer.

| Outcomes | №; studies (№; patients) | Research design | Factors that may decrease certainty of evidence | | | | | Effect per 100 patients tested | Test accuracy CoE |
| --- | --- | --- | --- | --- | --- | --- | --- | --- | --- |
|  |  |  | Risk of bias | Indirectness | Inconsistency | precision | Publication bias | Pre-test probability of ulcer/erosion % |  |
| **True positive** (Patients diagnosed with polyps/cancer) | 7 studies 6063681 patients | Case-control Study | Not serious | Not serious | Serious^[[9]](#footnote-9)^ | Not serious | Publication bias is strongly suspected^[[10]](#footnote-10)^ | 97 (92 to 99) | ⨁⨁◯◯ LOW |
| **False negative** (Patients misdiagnosed as having no polyps/cancer) |  |  |  |  |  |  |  | 3 (1 to 8) |  |
| **True negative** (People without polyps/cancer) | 7 studies 18127720 patients | Case-control Study | Not serious | Not serious | Serious^[[11]](#footnote-11)^ | Not serious | Publication bias is strongly suspected^[[12]](#footnote-12)^ | 98 (92 to 99) | ⨁⨁◯◯ LOW |
| **False positive** (Patients misdiagnosed as polyps/cancer) |  |  |  |  |  |  |  | 2 (1 to 8) |  |

1. Overall I2 for erosion/ulcer is 100%, which represent a high heterogeneity [↑](#footnote-ref-1)
2. According to the Deeks’ Funnel Plot Asymmetry Test, the P.value is 0.01, which indicate the exist of publication bias. [↑](#footnote-ref-2)
3. Overall I2 for erosion/ulcer is 100%, which represent a high heterogeneity [↑](#footnote-ref-3)
4. According to the Deeks’ Funnel Plot Asymmetry Test, the P.value is 0.01, which indicate the exist of publication bias. [↑](#footnote-ref-4)
5. Overall I2 for erosion/ulcer is 99%, which represent a high heterogeneity. [↑](#footnote-ref-5)
6. According to the Deeks’ Funnel Plot Asymmetry Test, the P.value is 0.02, which indicate the exist of publication bias. [↑](#footnote-ref-6)
7. Overall I2 for erosion/ulcer is 99%, which represent a high heterogeneity. [↑](#footnote-ref-7)
8. According to the Deeks’ Funnel Plot Asymmetry Test, the P.value is 0.02, which indicate the exist of publication bias. [↑](#footnote-ref-8)
9. Overall I2 for erosion/ulcer is 100%, which represent a high heterogeneity. [↑](#footnote-ref-9)
10. According to the Deeks’ Funnel Plot Asymmetry Test, the P.value is 0.00, which indicate the exist of publication bias. [↑](#footnote-ref-10)
11. Overall I2 for erosion/ulcer is 100%, which represent a high heterogeneity. [↑](#footnote-ref-11)
12. According to the Deeks’ Funnel Plot Asymmetry Test, the P.value is 0.00, which indicate the exist of publication bias. [↑](#footnote-ref-12)
